# Using motivational techniques to reduce cardiometabolic risk factors in long term psychiatric inpatients: a naturalistic interventional study

**DOI:** 10.1186/s12888-018-1832-6

**Published:** 2018-08-15

**Authors:** Petter Andreas Ringen, Ragnhild S. Falk, Bjørnar Antonsen, Ann Faerden, Asgeir Mamen, Eline B. Rognli, Dag K. Solberg, Egil W. Martinsen, Ole A. Andreassen

**Affiliations:** 1 Division of Mental Health and Addiction, Oslo University Hospital and NORMENT, KG Jebsen Centre, University of Oslo, Ullevål Hospital, P.O.Box 4956, 0424 Nydalen, Oslo Norway; 2 Oslo Centre for Biostatistics and Epidemiology, Oslo University Hospital, P.O.Box 4950, 0424 Nydalen, Oslo Norway; 3 Department of Psychiatry, Lovisenberg Diaconal Hospital, P.O.Box 4970, 0440 Nydalen, Oslo Norway; 4 Kristiania University College, P.O.Box 1190, 0107 Sentrum, Oslo Norway; 5 Division of Mental Health and Addiction, Oslo University Hospital, Ullevål Hospital, P.O.Box 4956, 0424 Nydalen, Oslo Norway; 6 Skjelfoss Psychiatric Center, Lukas Foundation and Center for Psychopharmacology Diakonhjemmet Hospital, Postboks 23, 0319 Vinderen, Oslo Norway; 7 Division of Mental Health and Addiction, Oslo University Hospital and University of Oslo, Ullevål Hospital, P.O.Box 4956, 0424 Nydalen, Oslo Norway; 8 NORMENT, KG Jebsen Centre, Oslo University Hospital, and University of Oslo, Ullevål Hospital, Building 49, P.O. Box 4956, 0424 Nydalen, Oslo Norway

**Keywords:** Cardiometabolic, Cardiovascular, Motivation, Intervention, Lifestyle, Psychosis, Schizophrenia, Inpatient, Physical activity

## Abstract

**Background:**

People with severe mental illness have markedly reduced life expectancy; cardiometabolic disease is a major cause. Psychiatric hospital inpatients have elevated levels of cardiometabolic risk factors and are to a high degree dependent of the routines and facilities of the institutions. Studies of lifestyle interventions to reduce cardiometabolic risk in psychiatric inpatients are few. The current study aimed at assessing the feasibility and effects of a lifestyle intervention including Motivational Interviewing (MI) on physical activity levels, cardiometabolic risk status and mental health status in psychotic disorder inpatients.

**Methods:**

Prospective naturalistic intervention study of 83 patients at long term inpatient psychosis treatment wards in South-Eastern Norway. Patients were assessed 3–6 months prior to, at start and 6 months after a life-style intervention program including training of staff in MI, simple changes in routines and improvements of facilities for physical exercise. Assessments were done by clinical staff and included level of physical activity, motivation, life satisfaction, symptom levels (MADRS, AES-C, PANSS, and GAF) as well as anthropometric and biochemical markers of cardiometabolic risk. A mixed model was applied to analyze change over time.

**Results:**

A total of 88% of patients received MI interventions, with a mean of 2.5 MI interventions per week per patient. The physical activity level was not increased, but activity level was positively associated with motivation and negatively associated with positive symptoms. Triglyceride levels and number of smokers were significantly reduced and a significant decrease in symptom levels was observed.

**Conclusions:**

The current results suggest that a simple, low cost life-style intervention program focusing on motivational change is feasible and may reduce symptoms and improve lifestyle habits in psychosis patients in long term treatment facilities. Similar programs may easily be implemented in other psychiatric hospitals.

**Trial registration:**

ClinicalTrials.gov. NCT03528278, date of registration: 05/16/2018 (retrospectively registered).

**Electronic supplementary material:**

The online version of this article (10.1186/s12888-018-1832-6) contains supplementary material, which is available to authorized users.

## Background

People with severe mental disorders carry a significantly higher risk for premature deaths due to cardiovascular disease [1]. Some of this increased risk seems to be linked to the genetic susceptibility of the disease itself [2], but several unhealthy lifestyle habits are also possible causes [1]. Tobacco smoking is highly prevalent [3], and has well-known detrimental effects on health and mortality, also in schizophrenia [4]. Many people with severe mental illness have unhealthy diets, often driven by poor economy or loss of interest in personal maintenance [5]. Side effects of especially the second generation antipsychotics are regarded as an important contributor to the increased risk for the metabolic syndrome in schizophrenia [6–8].

A balanced diet may reduce cardiovascular disease (CVD) risk factors [9–11]. Physical exercise has beneficial effects on CVD risk factors such as body mass index (BMI), and blood lipids [12] and may reduce mortality [13]. Improvement of physical fitness seems likely to reduce the elevated mortality in this patient group [14]. Exercise programs are feasible and may also improve mental well-being and overall outcome among patients with schizophrenia [15]. Increased physical exercise or improvements in dietary habits are thus possible strategies for reducing the increased mortality in schizophrenia.

There are, however, many barriers to life-style changes in the general population, and in addition several obstacles for changes specifically among patients with severe mental illness [16]. Some of these, such as negative symptoms and reduced initiative, are often part of the mental disorder itself. Many patients are ambivalent to making a change in their lifestyle habits and need help in their motivational process [17]. An increased focus on facilitating life-style changes related to cardiometabolic risk in the mental health care system would be of great importance.

Different motivational methods have been used for facilitating behavioural change in psychiatric settings [18, 19], with positive results for increased physical exercise in schizophrenia patients in outpatient and day care settings [20–23]. Motivational interviewing (MI) was originally developed for facilitating behavioral change in substance use disorders [24], but has spread to other areas of medicine where behavior change is crucial [25, 26]. Despite the accumulated evidence, comprehensive interventions aiming to improve physical fitness among patients with mental illness have only to a small degree been implemented in psychiatric clinics, and research on feasibility and effects of training programs in a hospital setting is scarce. To our knowledge no previous study has addressed the usefulness of MI for increasing physical activity or other lifestyle changes in severe mental illness in an inpatient setting. There is a need for low cost interventions in this area which are feasible to implement in daily clinical routines.

A project for improvement of lifestyle factors associated with cardiometabolic risk was carried out at several long term inpatient psychosis treatment wards in Southeastern Norway. Comprehensive clinical data at baseline confirmed high levels of cardiometabolic risk factors including obesity, smoking and unhealthy eating habits [27]. The current project aimed to answer the following research questions: How feasible is it to implement a simple lifestyle improvement program based on MI interventions targeting cardiometabolic health? Will level of motivation be associated with interventions or lifestyle changes? Further, will the intervention be associated with increased levels of physical activity, reduced cardiometabolic risk factors or improved mental health status?

## Methods

### Setting

The current study was carried out at The Oslo University Hospital (OUH) and the private inpatient psychiatric care facility Skjelfoss Psychiatric Centre - Lukas Foundation (SPC). At the OUH the two participating departments were in three different buildings at Gaustad in Oslo Municipality and at two sites at Dikemark in Asker Municipality, west of Oslo. The SPC is located in Hobøl Municipality south of Oslo. All departments provide inpatient treatment for long term psychiatric patients serving catchment areas in the South-East Health Region of Norway. The departments have the capacity to administer compulsory measures and included high security wards. The total number of beds was 116.

### Inclusion criteria

Inpatients, aged between 18 and 65 years and with DSM-IV diagnoses of schizophrenia, schizoaffective disorder, schizophreniform disorder, psychotic disorder NOS, bipolar I or bipolar disorder NOS. Exclusion criteria were established cognitive deficit (IQ < 70), brain damage, BMI < 17.5, inability to speak Scandinavian or English language. All participants had to be able and willing to give informed consent to participate in the study.

### Design

A project for lifestyle change was conceived of by the local administration and clinicians involving changing clinical routines at all wards relying on existing resources, e.g. local personnel performing all tasks. Interventions should be clinically relevant and should be possible to be transformed into permanent changes of practice. Practical obstacles for establishing an adequate control condition and ethical considerations ruled out a randomized, controlled study. The current study is thus a prospective observational study of patient related conditions before and after the introduction of a lifestyle enhancement program targeting cardiometabolic health, following as many patients as possible through their course of inpatient treatment.

**Fig. 1:**
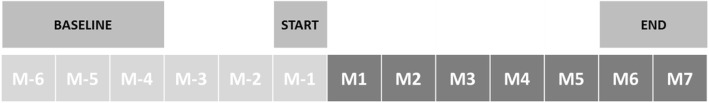
Design: Assessment periods at each ward. Months before (M-X) and after (MX) start of interventions. Dark grey: Intervention period

The intervention was preceded by *Start* assessments performed 0–4 weeks prior, and was followed by *End* assessments performed 6–7 months after the starting date for the interventions. The *End* assessments were compared to the assessments of the pre-intervention period (Fig. 1), which also included *Baseline* assessments performed 3–6 months prior, and thus served as a control condition.

Consecutive patients were included during a 12- month period which differed in onset between wards. Inclusion to the study at the first ward started in January 2013 and ended at the last ward in March 2015. Effort was made to have a continuous inclusion unbiased of patient related factors. The intervention was carried out at all wards and implemented at a date specified for each of the wards.

### Intervention

The intervention aimed at a life style change to reduce cardiometabolic risk. A user representative and the clinical staff at all participating wards were involved in the planning of the intervention, which consisted of three components:

1. *Motivational interventions*

Training in motivational interviewing (MI) was mandatory for all staff and was organized by certified instructors as a two-day intensive course with follow-up practical guidance. The course included lectures, discussion, role play and group guidance with the MI-expert. The first day of the course was held within a week before the defined starting day for interventions, the second within four weeks after. Patients were informed about the purpose and implications of the interventions. Motivational interventions for enhancing physical activity, dietary improvement and smoking cessation were applied in different settings: 1) Daily motivating interaction with patients was encouraged. All patients were offered at least 15 min structured MI per week, often performed during hikes in the countryside. In order to help adherence to protocol, staff used a daily recording form where type of MI-interventions, patients’ response to the MI-interventions and patients’ physical activity was recorded. This form is attached (in English translation) as supplementary material (Additional file 1). During all physical training activities, the staff focused on individual feedback and encouragement. 2) Adherence to treatment plans (see next component) would lead to instant positive verbal feedback and attention from staff (reinforcement), while adherence failure would receive neutral feedback (no reinforcement). 3) Staff would behave in a facilitating manner e.g. by changing to training clothes at times for assistance in physical activity. 4) Logbooks were given to every participant for personal logging of activities and results. 5) T-shirts with a logo and slogan were issued for promoting a sense of togetherness.

2) *Physical activity as part of individual treatment plans*

Patients were offered a comprehensive physical training program and participants and staff together made weekly individual treatment plans including a goal of 30 min physical exercise three times per week as a minimum. The physical exercise consisted of any continuous physical activity, e.g. walking, running, ball-play, cycling, weight lifting or gymnastics.

3) *Establishment of a basic infrastructure for physical activity and improved diet*

An infrastructure for physical activity enhancement was implemented by establishing a gym (weight lifting equipment and treadmill and/or stationary bicycles), a high intensity interval training group as well as low intensity walking/cycling groups at each site. The gym was manned by a physiotherapist at regular hours giving individual exercise advice. Programs for interval training were developed. Physiotherapists made regular visits to all wards in order to secure the quality of the programs and ensure implementation. Details about these programs are provided as an additional file (see Additional file 2). The infrastructure was made available at the intervention starting date. The intervention also included improvement in diet. During the project between-meals offerings of vegetables and fruit were introduced and sugar containing beverages were replaced with water or beverages with artificial sweeteners.

### Assessments at baseline only

Data was gathered from interviews and medical charts. Time of admission, smoking, and use of current psychopharmacological medication were recorded. Height was measured. The participants were diagnosed with the Structural Clinical Interview for DSM-IV for axis I disorders (SCID-I) [28]. More details about data collection is reported elsewhere [27].

### Assessments at baseline, start and end

Cardiometabolic risk factors were assessed according to established guidelines [29]. Weight was measured and Body Mass Index (BMI) was calculated. Waist circumference was measured with a horizontal tape measurement from the top of the right iliac crests. S-Glucose, HbA1c, Insulin, C-reactive Protein (CRP), vitamin D and fasting plasma lipids (Total Cholesterol, High Density Lipoprotein - LDL, Low Density Lipoprotein - LDL and Triglycerides –TG) were measured. Smoking habits were reported.

Level of physical activity was assessed as the product of the rating scores of the questions of frequency of sessions (never: 0, less than once a week: 0.5, weekly: 1, 2–3 times a week: 2.5, almost daily: 5), intensity of sessions (low effort: 1, moderate effort: 2, almost maximum effort: 3) and duration of sessions (less than 15 min: .10, 15–29 min: .38, 30–60 min: .75, more than 60 min: 1.0) of physical activity in the HUNT questionnaire [30–32]. Sedentary behavior was assessed by daily waking hours of inactivity.

We used the life satisfaction item from the HUNT study, categorizing life satisfaction on a 7-point scale from extremely discontent to extremely content. Self-esteem was assessed by the Rosenberg Self-Esteem Scale [33]. The level of psychotic symptoms was measured with The Positive and Negative Symptom Scale (PANSS) [34], depressive symptoms with the Montgomery Asberg Depression Rating Scale (MADRS) [35]. Global symptoms and psychosocial functioning were measured by the Global Assessment of Functioning Scale (GAF), the scores were split into scales of symptoms (GAF-S) and functioning (GAF-F) to improve psychometric properties [36]. Apathy was measured with the abridged clinical version of the Apathy Evaluation Scale (AES-C-Apathy) [37, 38].

Motivation was measured by applying two visual analog scales from 0 (minimum) to 10 (maximum) assessing perceived importance for change and readiness for change.

### Weekly assessments during the intervention period

Recordings were made of number of MI session per patient per week.

Diagnostics and symptom assessments were done by medical doctors and psychologists who were part of the regular staff and had participated in a training course in SCID and PANSS assessments and regularly diagnostic consensus meetings led by clinically well experienced specialists.

### Statistics

All analyses were done using the IBM Statistical Package for the Social Sciences (SPSS) version 25.0 (SPSS Inc., Chicago, IL, USA). Descriptive statistics were presented as frequencies, proportions, mean and median with range. Group comparisons were evaluated with independent sample *t*-tests and Chi square tests as appropriate. Bivariate analyses were performed with Pearson’s r and Spearman’s rho for normally distributed or skewed data, correspondingly.

Linear mixed model analyses were performed to estimate the fixed effects of time (Baseline, Start and End) on the different outcome (dependent) variables. Individual patients and time were set as random factors. No covariance structure was pre-specified (i.e. unstructured). After model fit, we estimated marginal means for time to explore differences between the time points. If significant changes by time were observed, a posteriori analyses were performed to explore the potential contribution of key variables with clinical interest. Thus, sex, BMI or use of antipsychotics was included as fixed effects, one at a time, in these analyses. Further, to explore the potential contribution of motivation, depressivity, positive and negative symptom status and institution for changes in physical activity, the following variables were included, one at a time, as fixed effects: motivation (importance and readiness), PANSS-positive symptoms, MADRS, AES-C Apathy and institution. A secondary analysis was performed, substituting physical activity by sedentary behavior, in order to study physical inactivity.

## Results

Of a total of 114 patients who met the inclusion criteria, 83 patients (73%) signed informed consent. Mean age was 40.1 years (*n* = 80), 57 of 82 (69.5%) were male, 57 (76.0%) had schizophrenia, 7 (10.7%) had schizoaffective disorder and 10 (13.4%) had bipolar disorder or other psychotic disorder. Median admission time prior to the intervention was 24 months (range 1–240), *n* = 31. At Start 43 of 50 patients (86.0%) used antipsychotic medication. Some patients had attenuated or absent positive psychotic symptoms at the time of inclusion. Due to variable staff resources, time constraints and sometimes non-compliance with assessment protocol, full datasets at all time points could not be obtained. There were no significant differences in assessments at Start between patients with or without assessments at Baseline. Levels of physical activity and motivation at both Start and End were obtained for 16 patients. Data on smoking at both Start and End were obtained for 40 patients. Weekly MI reports were obtained for 79 patients (95%) at week 1 (Start), but declined to 50% at week 13. An additional figure shows this in more detail (see Additional file 3: Figure S1). A total of *n* = 73 (88%) of the participants received MI-sessions during the intervention period. Mean MI sessions per patient per week during the 6 months follow-up was 2.5 (SD 2.3). An additional figure shows this in more detail (see Additional file 4: Figure S2). Uncorrected post hoc analyses showed that mean number of MI sessions per patient per week was 1.4 (SD 1.3) at OUS wards and 5.3 (SD 2.0) at SPC wards (*p* < .001).

The levels of physical activity increased from Start to End in 38% (6/16). Motivation levels at End were higher in the group with increase in physical activity; importance: 8.0 (SD 2.1) vs 4.9 (3.8), *p* = .056; readiness: 6.7 (1.4) vs 4.2 (4.1), *p* = .048. Among the smokers at Start, 17% (2/12) had quitted smoking at End, while among 28 initial non-smokers none started smoking (*p* < .001).

The mixed linear model analyses showed no statistically significant changes in estimated marginal means (EMMs) for physical activity or inactivity over time. There was a nominal trend for a reduction in sedentary behavior over time, although not statistically significant (*p* = .473). The reduction from Start to End of depressive symptoms (MADRS) was statistically significant (*p* = .009). There was a significant decrease of PANSS positive symptoms from Baseline to End (*p* = .006). The increase in self-esteem was borderline significant from Start to End (*p* = .051). There was a non-significant nominal *decrease* in both measures for motivation from Start to End (Importance: *p* = .473; Readiness: *p* = .179), while for the AES-C and the PANSS negative there were no apparent changes (Table 1).

**Table 1:** Linear mixed model analyses of clinical assessments at the Baseline, Start and End time points; estimation of marginal means (EMM) of time with standard errors (S.E)

Variables	N^a^	n	BASELINE		n	START		n	END	
			EMM	S.E.		EMM	S.E.		EMM	S.E.
Physical Activity Index	58	21	3.6	.6	51	3.0	.3	18	3.1	.5
Daily hours of inactivity	56	23	8.6	.7	47	8.0	.5	17	7.4	.7
Motivation, importance	72	55	7.0	.4	49	7.0	.4	19	6.4	.6
Motivation, readiness	72	54	5.9	.4	48	6.3	.4	19	5.5	.6
Self-esteem (Rosenberg)	60	21	25.7	.6	52	27.0	1.0	19	28.8	1.4
Life satisfaction	58	48	3.8	.2	18	3.5	.3	26	3.7	.2
GAF S	50	20	39.0	1.8	49	37.0	1.8	25	36.7	2.2
GAF F	50	20	37.8	1.6	49	36.1	1.6	25	38.1	2.2
PANSS positive	60	18	**22.3**	1.3	57	19.6	1.1	23	18.2	1.3
PANSS negative	60	17	20.1	1.7	56	21.3	1.6	23	19.9	1.2
PANSS general	60	17	43.3	2.3	56	39.6	1.5	23	38.2	1.8
MADRS	71	16	**13.5**	1.1	57	**12.8**	.9	23	10.3	1.1
AES-C Apathy	53	19	39.7	2.0	42	36.7	1.2	18	37.7	1.7
BMI, kg/m^2^	59	54	29.3	.9	34	30.0	1.0	17	29.4	0.9
Waist, cm	39	24	110.5	2.6	26	109.7	3.1	13	109.5	3.1
Glucose, mmol/L	57	21	5.6	.1	46	5.5	.1	17	5.5	.1
HbA1C, %	58	22	5.6	.2	48	5.4	.1	16	5.6	.1
Insulin, pmol/L	32	0	–	–	24	99.6	11.7	12	123.6	20.2
Cholesterol, mmol/L	57	22	5.1	.2	47	5.4	.1	17	5.2	.2
Trigycerides, mmol/L	57	22	1.8	.3	47	**2.2**	.2	17	1.5	.2
HDL-cholesterol, mmol/L	58	22	1.1	.1	48	1.4	.2	17	1.4	.1
LDL-cholesterol, mmol/L	57	20	3.0	.2	47	3.4	.1	16	3.3	.2
CRP, mg/L	25	21	3.5	.9	11	3.8	1.2	11	3.8	1.2
Vit DTOT, nmol/L	21	10	**46.7**	6.5	17	60.2	5.6	16	58.8	4.9

^a^: N of measures included in the linear mixed model. Bold numbers: Comparison with End yields significance at the .05 level. *GAF* Global Assessment of Functioning, *PANSS* Positive And Negative Syndrome Scale, *MADRS* Montgomery Asberg Depression Rating Scale, *AES-C Apathy* Apathy Evaluation Scale, *BMI* Body Mass Index, *HbA1C* Glycosylated Hemoglobin, *HDL* High Density Lipoprotein Cholesterol, *LDL* Low Density Lipoprotein, *EMM* Estimated Marginal Means, *S.E* Standard Error

There was a significant decrease in blood triglyceride levels after intervention (*p* = .002). For other blood lipids levels and CRP there were no clear trends for the intervention period (Start to End). There was a significant increase in D-vitamin levels from Baseline to End (*p* = .010) (Table 1). Adjusting for sex, BMI or use of antipsychotics at Start in the linear mixed model did not affect main findings.

Adjusted linear mixed model analyses showed a highly significant positive effect of motivation at Baseline (importance, *p* < .001, readiness, *p* = .011) and a significant negative effect of positive symptoms at Baseline on changes in levels of physical activity (*p* = .016). The MADRS and the AES-C at Baseline showed no significant effects on physical activity (Table 2). Similar analyses of sedentary behavior at baseline showed no significant effects of any of these potential covariates.

**Table 2:** Adjusted linear mixed model analysis of physical activity (PA Index) at the Baseline, Start and End time points; estimation of fixed effects

Variable		Coefficient	95% CI	p
Time	BL	0.49	−0.00 to 0.97	.050
	Start	−0.38	−1.53 to 0.76	.498
	End	(ref)		
Motivation, importance		0.23	0.14 to 0.32	<.001
Time	BL	0.37	−0.23 to 0.96	.199
	Start	−0.45	−1.78 to 0.89	.496
	End	(ref)		
Motivation, readiness		0.17	0.04 to 0.30	.011
Time	BL	0.50	.09 to 0.91	.022
	Start	0.24	−1.04 to 1.52	.701
	End	(ref)		
PANSS positive		−0.06	−0.11 to − 0.01	.016
Time	BL	0.53	−0.21 to 1.27	.136
	Start	0.28	−1.06 to 1.61	.664
	End	(ref)		
MADRS		0.03	−0.04 to 0.09	.394
Time	BL	0.78	−0.25 to 1.80	.111
	Start	−0.19	−1.45 to 1.07	.755
	End	(ref)		
AES-C Apathy		−0.06	−0.14 to 0.03	.170
Time	BL	0.55	−0.13 to 1.23	.099
	Start	−0.18	−1.32 to 0.96	.741
	End	(ref)		
Institution (OUH or SPC)		−0.32	−1.01 to 0.38	.365

*BL* Baseline, *PANSS* Positive And Negative Syndrome Scale, *MADRS* Montgomery Asberg Depression Rating Scale, *AES-C Apathy* Apathy Evaluation Scale, *CI* confidence intervals

## Discussion

The current study assessed a naturalistic intervention for lifestyle improvement including motivational interviewing to reduce cardiometabolic risk in a naturalistic long term inpatient ward setting. The study was observational, and relied on the institutions’ own staff for all interventions and assessments. The implementation of this low cost intervention was fairly successful, with an adequate number of MI-sessions, particularly in the first phase of the interventions. We found a significant reduction in triglyceride level and smoking after intervention. We did not detect any significant increase in mean activity level, but observed a significant decrease in depressive symptom level. We found change in physical activity to be positively associated with motivation and negatively associated with positive symptoms. To the best of our knowledge, the current findings are unique and highly clinically relevant as studies of low cost lifestyle interventions are scarce among psychiatric inpatient populations.

The findings of incomplete implementation of parts of the program may be taken as an indication that feasibility depended on available clinical resources and manpower. However, an evaluation of the health care system was beyond the scope of the present study. Thus, we have no systematic measures of budgets, work load, available staff resources at the long-term wards during the project period. However, we note that the long-term hospital SPC, with rural location, low patient turnover and no acute admissions, seems to have more MI sessions than the larger hospital OUH, with a high number of acute ward admissions per year. The design of the study with implementation by the local staff in regular clinical service, led to missing data. We have no indications that missing collection of data affected the quality of the assessments, or resulted in systematic bias in the data collected. The failure to improve the mean activity level and the lack of association between MI sessions and change in physical activity may suggest that the intervention was too weak or non-targeted. Previous studies suggest that quite comprehensive regimens are needed to make changes in physical activity level, and often provide modest effects [39–41]. However, sedentary behavior is increasingly considered as a more important target for interventions [42] and is possibly easier to improve. In fact, we observed a declining pattern in the inactivity level which did not reach statistical significance. Thus, the current findings indicate that there is a potential of low-cost cardiometabolic interventions in chronic inpatient settings. However, to support the notion that the current lack of significant effect is a type II error, more studies are needed.

We found that higher level of motivation was associated with improvement in physical activity level, but mean levels of neither of these improved during the intervention. A possible interpretation of these results is that interventions for physical activity enhancement only succeeded in targeting a subgroup of motivated patients. A proportion of patients seemed to reduce motivation during the project period, although not significant. This seems to reason with non-systematic evaluation of the registered individual patients’ reports suggesting that some psychological “saturation” effects might have been at play. Unfortunately, due to low N it was not possible to explore changes in symptoms or motivation across groups with or without improvement in physical activity.

Two patients quitted smoking during the interventions, and none started. It is conceivable that this highly clinically meaningful life style change is attributable to the staff’s motivational efforts which included smoking cessation. Smoking cessation may reduce the metabolism of antipsychotics and thus increase the effect of medication. This can potentially affect both activity level and symptom load. However due to the low number we were not able to control for this possible association. Further, we found that triglyceride level was significantly reduced from Start to End independently of BMI or use of antipsychotics, and there was no increase in other lipid levels. However, the change in triglyceride level was not associated with change in activity level, and this result must be interpreted with caution. We could speculate that the change is related to improved diet, which is previously shown to improve lipid levels in schizophrenia patients [43]. Changing diet in a hospital setting depends on motivation both in patients and staff, which was a goal of the present intervention.

Level of depression was significantly decreased from Start to End, which suggests that it was independent of the general symptom improvement during hospitalization. Decreasing depression level after interventions for enhancement of physical activity has been shown in previous meta-analyses [39, 44]. As we did not detect any increase in mean activity level, it is possible that the observed reduction in the MADRS score could be associated directly with the motivational work [45] and a potential change in ward atmosphere [46]. It is of interest that improving mood is an important motivator for physical exercise in SMI [16]. A previous analysis of the Baseline data found high level of depression to be associated with low level of physical activity [27]. However, the level of depression was not associated with change in physical activity in the current intervention study.

The finding of a decrease in positive symptoms was only significant from Baseline to End and could hence not be associated with the intervention specifically. However, reduced level of positive symptoms was associated with an increase in physical activity. A reduction in positive symptoms during exercise interventions has been shown in other studies [39, 47]. This might have clinical importance.

The main strength of the study is the naturalistic design with comprehensive clinical descriptions and the assessments of changes in “real-life” psychosis-treatment hospital wards. We argue that this design has important advantages for representativity and translational value, and our methods and experiences could be useful for other lifestyle enhancement projects. However, as the design was not experimental with e.g. a control group, there are limitations in interpreting effects of the interventions. There were incomplete data in the time series due to instability in data gathering resources of the clinical staff in the project period. This gave a risk for type II errors and limited the testing of hypotheses in subgroups. Bias could be introduced if there were any systematic selection of patients for assessments or missing data. However, we have tested for differences associated with missing data, and found no clear bias. We have no indications that other selection mechanisms were at play, but bias cannot be ruled out. Site or ward specific differences could also affect the results, but we had not statistical power for evaluating site-dependent factors. The mixed model analyses made it possible to test hypotheses of datasets with incomplete time series with a high retention of statistical power. The study of change in a naturalistic design made it impossible to compare change in a control group in the same time period. We were thus not able to control for effects related to time, something that is a concern in a setting of active treatment. By comparing the change in the intervention period to the pre-intervention period, we were nevertheless able to make some assessments of the contributions of time as a factor. Further, it can be argued that dramatic changes over some months in the intervention period are not expected in this population of predominantly chronically ill patients where over 50% had more than 2 years duration of admission at the time of inclusion. We did not have records of the staff’s attitudes and behavior or ward atmosphere, hence, potential changes in these factors associated with the interventions could not be controlled for. Another limitation is that we have no objective measurements of level of physical activity.

## Conclusions

In conclusion, the current study shows that a combined intervention for lifestyle change, with emphasis on motivational work is feasible in psychiatric inpatients settings, and may have positive effects on psychiatric symptom level as well as on lifestyle factors related to cardiometabolic risk. The study highlights both the importance of motivational factors in lifestyle change and the need for evidence based practices to guide milieu therapeutic interventions at inpatient hospital wards. The interventional program described in the current study relied on existing staff resources for the actual patient interventions and could easily be implemented elsewhere, provided motivated staff with necessary resources.

Viewing a psychiatric hospital as a place of retention is still a reality in minds, routines and legislations, and staffing of wards will often be more focused on avoiding unacceptable risks than on the improvement of clinical practice. A psychiatric hospital environment, however, has opportunities for the involvement of both users and staff in the planning and provision of better services with potential positive impact on highly important aspects of health.

## Additional files

Additional file 1: Daily entry form intervention period. (DOCX 15 kb)

Additional file 2: Detailed description of the 4X4 high intensity (HIT) training and the “Hill Run”. (DOCX 17 kb)

Additional file 3: **Figure S1.** Recordings of MI (n) per week after intervention start at each ward. Legend: x-axis: weeks counting from start of intervention. (DOCX 107 kb)

Additional file 4: **Figure S2.** Mean MI interventions per participant per week after intervention start at each ward. Legend: x-axis: weeks counting from start of intervention. (DOCX 101 kb)

## Abbreviations

AES-C Apathy: Apathy Evaluation Scale
BMI: Body Mass Index (BMI)
CVD: Cardiovascular disease
DSM: Diagnostic and Statistical Manual of Mental Disorders
EMM: Estimated Marginal Means
GAF: Global Assessment of Functioning
HbA1C: Glycosylated Hemoglobin
HDL: High Density Lipoprotein Cholesterol
LDL: Low Density Lipoprotein Cholesterol
MADRS: Montgomery Asberg Depression Rating Scale
MI: Motivational Interviewing
NOS: Not Otherwise Specified
OUH: Oslo University Hospital
PANSS: Positive And Negative Syndrome Scale
S.E: Standard Error
SCID: Structured Clinical Interview for DSM-IV Axis I Disorders
SPC: Skjelfoss Psychiatric Centre - Lukas Foundation
