# Supplementary material for: Using motivational techniques to reduce cardiometabolic risk factors in long term psychiatric inpatients: a naturalistic interventional study
Source: BMC Psychiatry. 2018 Aug 15;18:255. doi: 10.1186/s12888-018-1832-6 (PMC6094458; doi:10.1186/s12888-018-1832-6)
Supplement: Supplementary file 1 — Daily entry form intervention period. (DOCX 15 kb) [file 12888_2018_1832_MOESM1_ESM.docx]

**Additional file 1: DAILY ENTRY FORM INTERVENTION PERIOD**

Date:........................... Shift (Night/Day/Evening):...........

Patient: ............................................................................

**My MI-interventions**

| Type of intervention | Number | Purpose | Content/Quote |
| --- | --- | --- | --- |
| Open questions |  |  |  |
| Reflections |  |  |  |
| Recaps |  |  |  |
| Confirmations |  |  |  |

**The patient’s utterances on physical activity**

| Type of utterance | Number | Content/Quote |
| --- | --- | --- |
| Resistance |  |  |
| Change talk |  |  |

**Patient’s physical activity**

| Type of activity | Number/amount | Content/Intensity |
| --- | --- | --- |
| Walk/Hike |  |  |
| Training |  |  |
