# Supplementary material for: Using motivational techniques to reduce cardiometabolic risk factors in long term psychiatric inpatients: a naturalistic interventional study
Source: BMC Psychiatry. 2018 Aug 15;18:255. doi: 10.1186/s12888-018-1832-6 (PMC6094458; doi:10.1186/s12888-018-1832-6)
Supplement: Supplementary file 2 — Detailed description of the 4X4 high intensity (HIT) training and the “Hill Run”. (DOCX 17 kb) [file 12888_2018_1832_MOESM2_ESM.docx]

**SUPPLEMENT: Detailed description of the 4X4 high intensity (HIT) training and the “Hill Run”**

4X4 HIT training

Interval training was performed on treadmills using pulsemeters after a warm-up period of 5-10 minutes at 70 % of maximum heart rate (MHR). Each of the four intervals had duration of 4 minutes with an effort of 85-90 % of MHR. Between intervals there were brakes with lower intensity of about 70 % of MHR. For several patients an inclination of the band of 3-4 % was used.

Hill Run

MHR was calculated for the participants before start. Participants used pulsemeters. The warm-up period consisted of 5-10 minutes jogging at 70 % of MHR. 10 runs were performed running up a steep hill of about 150m with a brake after 5 runs. The brake lasted for about 5 minutes in order to avoid build-up of lactate in the muscles and was used for oral feed-back from the staff. On each run every participant was closely followed up and encouraged in order to achieve an intensity of 85-90 % MHR. Everyone ran as fast as they could and jogged or walked back down the hill after each run.

The Hill Run was easy to perform as there was little requirement for equipment, but demanded intense motivation work. Several patients got highly motivated.
