# Supplementary material for: Using motivational techniques to reduce cardiometabolic risk factors in long term psychiatric inpatients: a naturalistic interventional study
Source: BMC Psychiatry. 2018 Aug 15;18:255. doi: 10.1186/s12888-018-1832-6 (PMC6094458; doi:10.1186/s12888-018-1832-6)
Supplement: Supplementary file 3 [file 12888_2018_1832_MOESM3_ESM.docx]

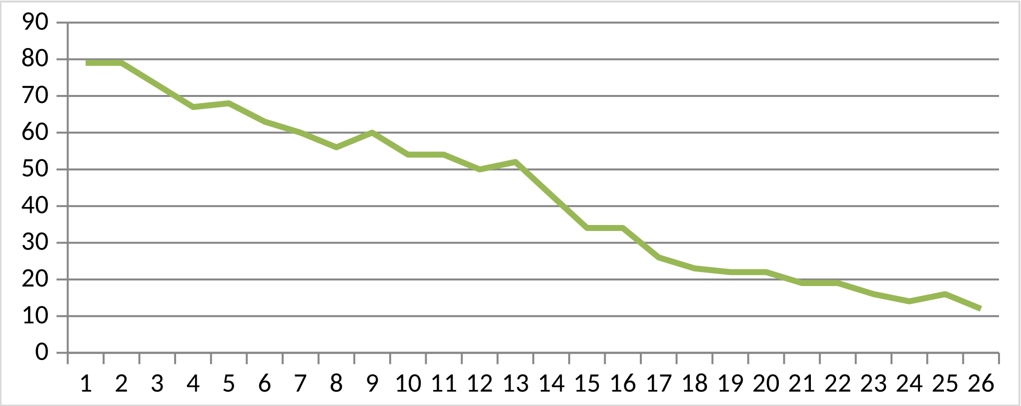

90
80
70
60
50
40
30
20
10
0
1 2 3 4 5 6 7 8 9 10 11 12 13 14 15 16 17 18 19 20 21 22 23 24 25 26
